# The role of GSK-3 in mood disorders: Preliminary data from an experimental study

**DOI:** 10.1192/j.eurpsy.2021.1025

**Published:** 2021-08-13

**Authors:** G. Di Salvo, G. Rosso, E. Hoxha, E. Teobaldi, I. Balbo, F. Tempia, G. Maina

**Affiliations:** 1 Department Of Neurosciences Rita Levi Montalcini, University of Turin, Turin, Italy; 2 Neuroscience Institute Cavalieri Ottolenghi (nico), Department of Neuroscience, Turin, Italy

**Keywords:** Mood disorder, Biomarkers, GSK-3, Differential Diagnosis

## Abstract

**Introduction:**

The identification of potential biomarkers is crucial to improve the management and treatment of mood disorders. Glycogen synthase kinase-3 (GSK-3) is a multifunctional enzyme with an important role in the etiology of mood disorders. Recent findings suggested GSK-3 as a putative biomarker in mood disorders.

**Objectives:**

The aims of the study are: - to evaluate GSK3 as potential biomarker for differential diagnosis (MDD and BD); - to analyze the regulation of GSK3 by psychopharmacological treatments.

**Methods:**

Patients included fulfill the following criteria: (a) principal diagnosis of MDD or BD (DSM-5); (b) age ≥ 18 years; (c) drug-free for at least 4 weeks before the inclusion. For each patient included a healthy control is enrolled, matched by gender and age. All included subjects at the study entry point (t0) are assessed through: - semistructured clinical interview and clinical rating scales (Hamilton Depression Rating Scale, Hamilton Anxiety Rating Scale; Young Mania Rating Scale, Clinical Global Impression) - blood draw, to measure GSK-3 levels Patients with MDD or BD are assessed again after 1 week (T1) and after 2 month (T2) of specific pharmacological treatment.

**Results:**

So far, we enrolled 16 patients and 16 healthy controls. The enrollment is still ongoing.

**Conclusions:**

We expect to find GSK-3 levels differently expressed between healthy controls, patients with DDM and patients with BD. This finding would be crucial as it could contribute to the improvement of differential diagnosis. Moreover, we expect to observe a change in GSK-3 levels after psychopharmacological treatments.

